# COVID-19 in New Mexico Tribal Lands: Understanding the Role of Social Vulnerabilities and Historical Racisms

**DOI:** 10.3389/fsoc.2020.610355

**Published:** 2020-12-22

**Authors:** Aggie J. Yellow Horse, Nicholet A. Deschine Parkhurst, Kimberly R. Huyser

**Affiliations:** ^1^School of Social Transformation, Arizona State University, Tempe, AZ, United States; ^2^Department of Sociology, University of British Columbia, Vancouver, BC, Canada

**Keywords:** COVID-19, Indigenous Peoples, Tribal lands, historical racisms, Social Vulnerability Index

## Abstract

The Coronavirus 2019 (COVID-19) pandemic has disproportionally affected Indigenous Peoples. Unfortunately, there is no accurate understanding of COVID-19's impacts on Indigenous Peoples and communities due to systematic erasure of Indigenous representation in data. Early evidence suggests that COVID-19 has been able to spread through pre-pandemic mechanisms ranging from disproportionate chronic health conditions, inadequate access to healthcare, and poor living conditions stemming from structural inequalities. Using innovative data, we comprehensively investigate the impacts of COVID-19 on Indigenous Peoples in New Mexico at the zip code level. Specifically, we expand the U.S. Centers for Disease Control and Prevention's Social Vulnerability Index (SVI) to include the measures of structural vulnerabilities from historical racisms against Indigenous Peoples. We found that historically-embedded structural vulnerabilities (e.g., Tribal land status and higher percentages of house units without telephone and complete plumbing) are critical in understanding the disproportionate burden of COVID-19 that American Indian and Alaska Native populations are experiencing. We found that historically-embedded vulnerability variables that emerged epistemologically from Indigenous knowledge had the *largest* explanatory power compared to other social vulnerability factors from SVI and COVID-19, especially Tribal land status. The findings demonstrate the critical need in public health to center Indigenous knowledge and methodologies in mitigating the deleterious impacts of COVID-19 on Indigenous Peoples and communities, specifically designing place-based mitigating strategies.

## Introduction

The Coronavirus 2019 (COVID-19) pandemic has disproportionally affected Indigenous Peoples globally and in the United States. COVID-19 has spread in Indian Country^1^—general description of Native space and place in the United States, and is inclusive to the Native Nations that occupy the spaces—through pre-pandemic mechanisms ranging from disproportionate chronic health conditions, inadequate access to healthcare, and poor living conditions generated from structural inequalities. While the current pandemic has exacerbated inequalities and health inequities,^2^ which were already critical issues pre-pandemic, the true estimations of the magnitude of such effects continue to be challenging due to long-standing and systematic erasure of Indigenous representation in data and race misclassification (Kukutai and Taylor, 2016; Yellow Horse and Huyser, 2020). That is, despite the critical importance of reliable and accurate COVID-19 statistics in mitigating the impacts of COVID-19, public health data often completely omit Indigenous Peoples or misclassify them as “others” by aggregating Indigenous Peoples with other numerically small populations. Even as of late August 2020, roughly 6 months into the pandemic, less than half of U.S. states report race-specific COVID-19 information for American Indian and Alaska Native (AIAN, hereafter) persons (Hatcher et al., 2020). Where such data is available, it shows a small glimpse of disproportionate impacts of COVID-19 that AIAN persons are experiencing. Overall, AIAN persons are 3.5 times more likely to experience COVID-19 than non-Hispanic white individuals (Hatcher et al., 2020), but the rates of COVID-19 for AIAN persons substantially vary across the United States. For example, in New Mexico, although the AIAN population represent only 9.6% of the state's total population, they account for nearly 50.8 and 60.3% of the state's total confirmed COVID-19 cases and deaths, as of August 2020 (New Mexico Department of Health, 2020). Notable, the AIAN population account for the largest proportion of the state's total confirmed COVID-19 cases, but they are also most likely to die from COVID-19 related complications.

Despite these data challenges, amplifying the experiences of Indigenous Peoples and communities during COVID-19 is critical on multiple accounts. First and foremost, stories on experiences of Indigenous Peoples and communities during the pandemic is yet another powerful testament to Indigenous resilience and the strengths of Tribal Sovereignty. Despite the magnitudes and multitudes of historical injustices Indigenous Peoples experienced from forced assimilation and separation of families through Indian boarding schools (Lomawaima, 1995), environmental contamination due to resource extraction (Hoover et al., 2012), institutional marginalization (Wilkins and Lomawaima, 2001), and violations of treaty agreements (Prucha, 1994); Indigenous Peoples are “still here” as the past, present, and future stewards of the lands. Furthermore, the experiences of Indigenous Peoples and communities during the pandemic shed light on how social inequalities are the consequences of historical racisms and are central to contemporary health inequalities. Identifying racism as the fundamental cause of health inequalities and inequities for Indigenous Peoples is critical because it means that policy solutions to mitigate the effects of COVID-19 should not only focus on addressing the mechanisms but also racism itself (Link and Phelan, 1995).

## Social Vulnerability Index and COVID-19

The Social Vulnerability Index (SVI, hereafter) was first developed by a geographer as a tool for natural disaster emergencies and evacuation planning (Cutter et al., 2003), and has been adapted and implemented by the United States' Centers for Disease Control and Prevention (CDC, hereafter) “to help local officials identify communities that may need support in preparing for hazards; or recovering from disaster” (Flanagan et al., 2011, 2018; Centers for Disease Control and Prevention, 2018). The central idea of the SVI is to identify more socially vulnerable areas, based on multiple indicators, for the implementation of *place-based* intervention to facilitate recovery from disaster. That is, by identifying places to employ the intervention effort officials can maximize its efficiency and impacts. The CDC's SVI is calculated by using 15 variables from Census data representing four distinct dimensions of social vulnerability (Flanagan et al., 2011, 2018): (1) Socioeconomic status vulnerability, (2) household composition and disability vulnerability, (3) minority status and language vulnerability, and (4) housing and transportation vulnerability.

Since the early phase of the pandemic, SVI has gained considerable popularity with different government entities and non-profit organizations to assess the spatial variations of disproportional impacts of COVID-19 in the United States. For example, Social Progress Imperative, a non-profit organization, launched an online platform documenting SVI and COVID-19 rates for 500 cities in the United States (Social Progress Imperative, 2020). Researchers in the United States already used the SVI to assess the impact of social vulnerability on COVID-19 outcomes nationally at the county level (Karaye and Horney, 2020; Nayak et al., 2020); and regionally at the zip code level to look at the disproportionate impacts on Black people, in particular during the early phase of the pandemic (most COVID-19 data dated April, 2020) (Amram et al., 2020; Gaynor and Wilson, 2020; Kim and Bostwick, 2020). These studies found that areas with high percentage of Black people were highly correlated with social vulnerabilities, and suggest that existing social vulnerabilities exacerbated COVID-19 outcomes for Black people (Gaynor and Wilson, 2020; Kim and Bostwick, 2020).

Despite the popularity of SVI as a potential tool for mitigating COVID-19 efforts, there are two critical questions that must be addressed. First, it is important to assess whether and how CDC's SVI is a useful policy tool for COVID-19 mitigation efforts beyond its original intended use for natural disaster emergencies and evacuation planning. That is, SVI as a tool for identifying highly vulnerable places for natural disaster might not be sufficient in identifying highly vulnerability places for COVID-19. For example, SVI does not include factors that are directly associated with spread and treatment of infectious diseases such as population density and percent of population without access to health insurance (Hu et al., 2013). Second, even we assumed the effectiveness of SVI in COVID-19 mitigation efforts in general; whether and how SVI can help the mitigation efforts for Indigenous Peoples, places and communities remains unclear. For example, in early data analysis of Arizona and New Mexico counties that fall within the Navajo Nation compared with neighboring counties data showed that while counties on and off the Navajo Nation both have high vulnerability scores, the score alone does not provide insight into why COVID-19 spread quickly in Navajo communities (at the time of that data's publishing) (Eisenberg, 2020). Calculation of SVI accounts for a percent of racial and ethnic minority without specificity, it likely favors numerically large racialized and minoritized groups. Due to small population sizes of Indigenous Peoples, and “relatively low level of racial residential segregation from non-Hispanic whites” especially in larger geographic scales (Byerly, 2019), Indigenous Peoples are likely to be systematically marginalized in place-based COVID-19 mitigation efforts through becoming invisible by exclusionary population aggregation (Monmonier, 2018). Furthermore, SVI does not account for other important structural inequality factors stemming from historical injustices that are critical and specific for experiences of Indigenous Peoples and communities.

## Historical Racisms, Tribal Land Status, and Abandoned Uranium Mines

Historically-embedded structural racism is a fundamental cause of health inequities in the United States (Phelan and Link, 2015; Boyd et al., 2020); and contributes to the widening of racial health inequities through its impacts on social conditions as pre-existing risk factors (Garcia et al., 2020; Pirtle, 2020). Framing historically-embedded structural racisms as the fundamental cause means that the significant relationship between racism and health outcomes would persist over time despite intervening mechanisms that appear to improve health (Phelan and Link, 2015). Explicitly considering the role of racism in understanding how COVID-19 is disproportionately affecting Indigenous Peoples and communities is also important for designing policy solutions to address the racism directly rather than its mechanisms (Hicken et al., 2018; Cogburn, 2019). By focusing on structural factors above and beyond individual characteristics is particularly important to refute the notion of “individualization of health” that solely puts the responsibility of health on the individuals by focusing individual behavior modification (e.g., frequent hand wash) (e.g., Mendenhall, 2016) without considering the structural factors that may influence individual behaviors (e.g., lack of access to safe water at home for frequent hand wash).

To successfully mitigate the disproportionate impacts of COVID-19 on Indigenous Peoples and communities where racism is a fundamental cause. It is not only critical to address public health challenges in Tribal public health such as the shortage of personal protective equipment (i.e., intervening mechanisms), but also to directly address the violations of treaties and other agreements (i.e., racism). In this light, Tribal land status, AIAN legal or statistical geographic areas, is important to explicitly consider as a proxy measure of systematic racism (Thornton, 1987). In New Mexico and for this study, Tribal lands are inclusive of the legal federally recognized American Indian reservations, off-reservation trust land, and tribal subdivisions (U.S. Census Bureau, 2002). That is, the characteristics of Tribal land is not simply an aggregation of individuals who occupy the land, but embody the lasting legacy of historically-embedded structural racism.

Tribal lands do not simply reflect choices and preferences of individuals on Tribal lands, but it reflects the lasting impacts of multiple historical racisms including racist reproductive policies to surveillance of reproductive health of Indigenous Peoples directly affecting the limited access to reproductive health services (Lawrence, 2000; Arnold, 2014; Gurr, 2014; Theobald, 2019) and environmental contamination influencing the health of Indigenous Peoples (Hoover, 2018). For example, after the passage of Family Planning Services and Population Research Act of 1970, it is estimated that nearly one in four Native American women of childbearing age were sterilized without consent until 1976 (Lawrence, 2000; Gurr, 2014). At the same time, the Hyde Amendment (first passed in 1976 then not amended until 1993) which made the use of federal funds for abortion services illegal denied Native American women's access to reproductive health services through lack of funding to Indian Health Services (Arnold, 2014). There is also substantial evidence on how residing close to abandoned uranium mines are associated with various reproductive physiological damages (Harmon et al., 2017). Tribal land status is also related to the lack of Internet access as Tribes have unique geopolitical and geophysical terrain influenced by colonization, cultural practices, sovereignty and Tribal governance (Warner, 1998; Monroe, 2002). That is, while access to information and communication technology, such as the Internet, depends on aspects of Tribal sovereignty there are other external obstacles such as federal policies, statutory and regulatory requirements, and historically overlooked and underfunded Internet infrastructure (Howard, 2019; Morris and Howard, 2020).

Furthermore, presence of abandoned uranium mines —as a legacy of environmental racism for Indigenous Peoples—is critical in understanding the disproportionate impacts of COVID-19 on Indigenous Peoples and communities. Presence of abandoned uranium mines is not only associated with environmental contamination (e.g., high rates of toxin exposure) and lack of safe water (Gilliland et al., 2000; Credo et al., 2019), but it is also closely related to structural inequalities (e.g., poor social conditions) (Deschine Parkhurst et al., 2020). That is, areas with abandoned uranium mines may also have a higher percentage of households without complete plumbing and access to safe water that are related to COVID-19 mitigating efforts. Research systematically documents the lasting deleterious effects of mining and abandoned uranium mines on health of Indigenous Peoples and communities through multiple interconnected mechanisms (Lewis et al., 2017). Whether the presence of abandoned uranium mines also have lasting indirect spillover effects on COVID-19 rates is an important question as historically-embedded structural racism affects the health of Indigenous Peoples and communities through multiple intervening mechanisms (Phelan and Link, 2015). Acknowledging the importance of the lasting legacy of abandoned uranium mines on the health of Indigenous Peoples, the National Institute of Environmental Health Sciences within the National Institute of Health awarded two Navajo scientists to examine the relationships between abandoned uranium mines and lack of access to clean water which can have implications for the increased COVID-19 rates on Diné Bikéyah (Navajoland) (Saffron, 2020).

## The Present Study

Confronted with challenges in data availability along and the critical need to examine the impact of the current pandemic with racism as a fundamental cause, we assemble an innovative data to comprehensively investigate the impacts of COVID-19 on Indigenous Peoples in New Mexico. New Mexico is home to 23 Native Nations including 19 Pueblos and three Apache tribes. New Mexico has the fourth largest presence of single-race AIAN peoples in the United States with 7.4% of the total AIAN population in the United States (199,896 out of 2,707,577). However, except for Alaska where the AIAN population accounts for 14.4% of the State's total population, New Mexico has the highest proportion of AIAN as the State's total population with 9.6% of the New Mexico population self-identified as AIAN (for example, the percentages are 7.5% in Oklahoma and 4.5% in Arizona, respectively) (U.S. Census, 2019). Furthermore, New Mexico has released COVID-19 related information for all their zip codes without suppressing the information for Tribal lands (in contrast, Arizona had not released any COVID-19 related information on Tribal lands). The data has shown disproportionate effects of COVID-19 on AIAN Peoples in New Mexico (New Mexico Department of Health, 2020).

**Figure 1:**
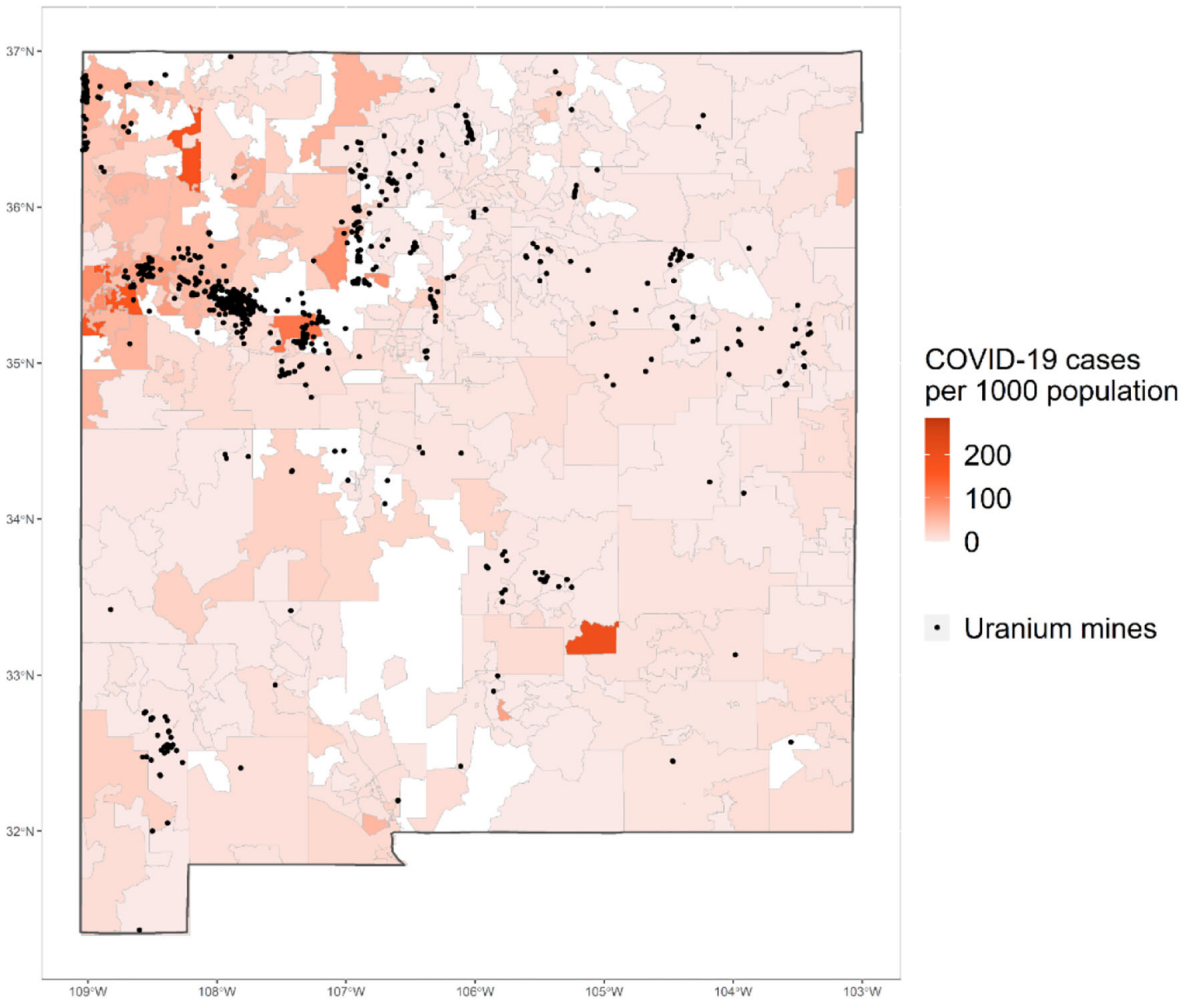
Confirmed COVID-19 cases per 1,000 and abandoned uranium mines in New Mexico.

Guided by Hózhó wisdom of Navajo people that emphasize the importance of interconnectedness and whole-system (Powell and Curley, 2008; Kahn-John and Koithan, 2015), we look at the relative impacts of multiple social vulnerability factors driven from the CDC's SVI (Flanagan et al., 2011, 2018) as well as the relative impacts of *historically-embedded* vulnerability factors that are important for Indigenous Peoples on the population-adjusted confirmed COVID-19 cases at the zip code- level in New Mexico. Grounded in an Indigenous research paradigm (Hart, 2010), we center Indigenous knowledge and methodologies. Particularly, we recognize the fluidity of knowledge derived from being Indigenous researchers who have lived on and in Indigenous lands in New Mexico, and we are committed to generating research that values and respects Indigenous communities (Hart, 2010; Smith, 2013). Thus, we pay distinct attention to the Tribal land status, presence of abandoned uranium mines, and lack of access to telephone, Internet and complete plumbing—to center the importance of structural inequalities stemming from historical racisms on the lands where Indigenous Peoples live and steward. Explicit inclusion of historically-embedded vulnerability factors as well as emerged epistemologically from Indigenous knowledge through stories and perpetual experiences of the authors. In addition to data availability, New Mexico is an ideal place for this case study as the lasting deleterious health effects of abandoned uranium mines have been well-documented in New Mexico (e.g., Jones, 2014). Substantial amounts of abandoned uranium mines exist on Diné Bikéyah on northwestern New Mexico, as well as throughout the states. Figure 1 shows the map of confirmed COVID-19 cases per 1,000 population (as of August 9, 2020) and distribution of abandoned uranium mines.

## Materials and Methods

### Data

To assess the relative impacts of various indicators of social vulnerability and structural inequalities from historical injustices on the confirmed COVID-19 cases in New Mexico; we assembled the unique data from multiple sources. Our dependent variable (i.e., the COVID-19 confirmed cases per 1,000 population) comes from the New Mexico Department of Health COVID-19 Dashboard (New Mexico Department of Health, 2020). Population information, indicators of social vulnerability as defined by the SVI which is widely used by the CDC (Flanagan et al., 2011, 2018), and structural inequality indicators come from the latest 5-year estimates from the American Community Survey 2014–2018 (U.S. Census, 2019). Lastly, information about the Tribal land status and presence of abandoned uranium mines come from the ESRI New Mexico Tribal lands shapefile and the New Mexico uranium mines shapefile originating from the U.S Geological survey from the U.S. Department of the Interior (U.S. Department of the Interior, 2011). All data are linked using zip code as the common geographic identifier.

### Measures

We created the dependent variable, confirmed COVID-19 cases per 1,000 population for each zip code, by dividing the cumulative counts (as of August 9, 2020) of confirmed COVID-19 cases by the zip code total population multiplied by 1,000. We log-transformed the dependent variable to meet the parametric requirement for normality, consistent with previous studies on COVID-19 (Karaye and Horney, 2020).

We included 15 variables from the four dimensions of vulnerability in the SVI used by the CDC (Flanagan et al., 2011, 2018). For the *socioeconomic status vulnerability* dimension of the SVI, we included four variables: percent of population living below poverty, percent of population who are unemployed, logged per capita income, and percent of population without a high school diploma. For the *household composition and disability vulnerability* dimension of the SVI, we included four variables: percent of children population aged under 18, percent of elder population aged 65 and older, percent of population with a disability, and percent of single-parent household. For the *minority status and language vulnerability* dimension of the SVI, we included two variables: percent of minority (i.e., who are not non-Hispanic white) and percent of population speaking English less than “well.” For the *housing and transportation vulnerability* dimension of the SVI, we included five variables: percent of housing units that are large apartment buildings (i.e., more than 10 units per structure), percent of housing units that are mobile homes, percent of crowded households (i.e., having more than one occupant per room), percent of population without a vehicle, and percent of population living in group quarters. In addition, we added two variables to capture *COVID-19 related vulnerability*: logged population density and percent of population without insurance.

**Figure 2:**
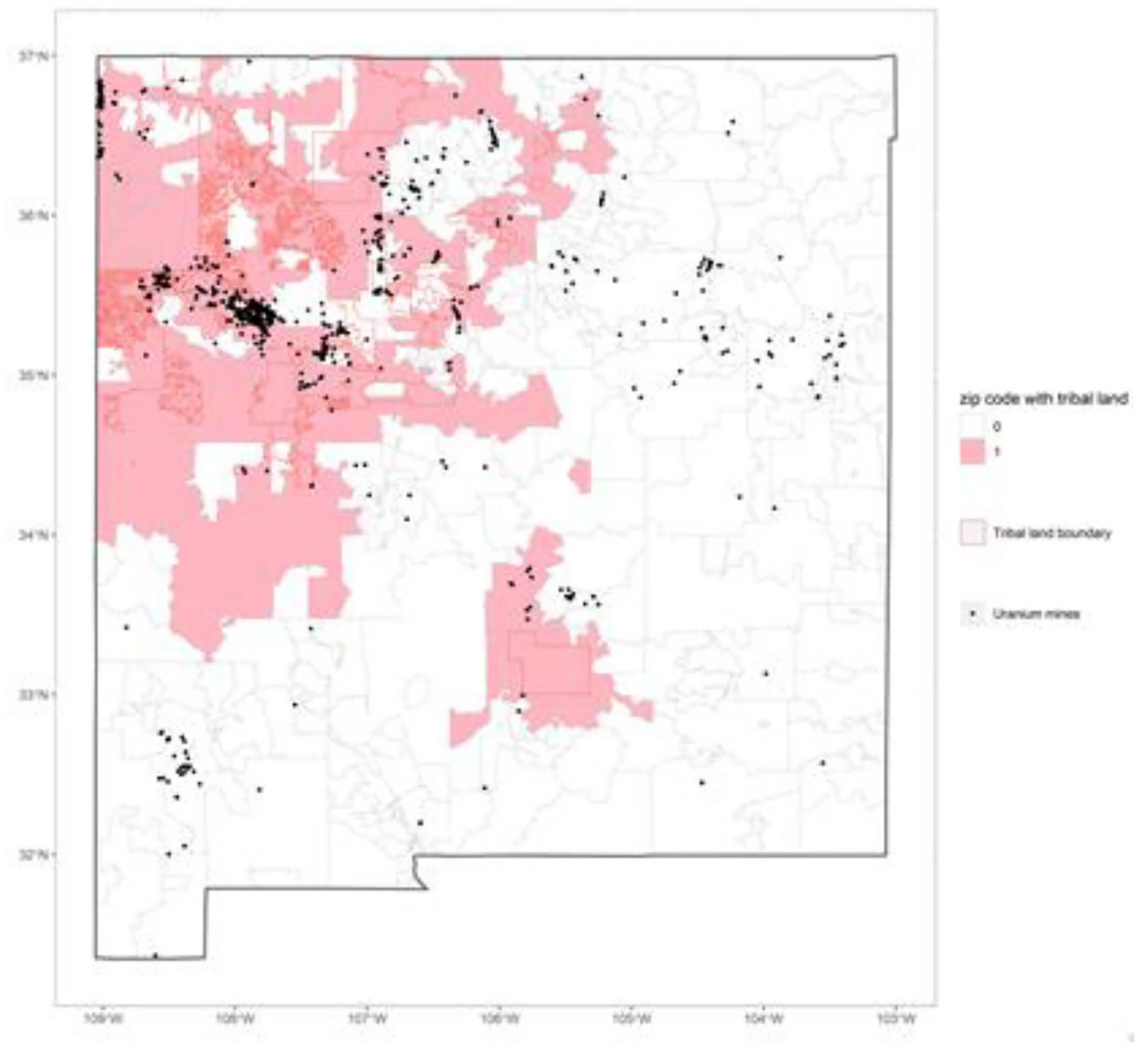
Zip codes containing Tribal lands and abandoned uranium mines in New Mexico.

We included five *historically-embedded vulnerability* variables: percent of housing units without telephone, percent of housing units without Internet, percent of housing units without complete plumbing, Tribal land status, and presence of abandoned uranium mines. To create the dichotomous indicator of whether the zip code include any Tribal lands, we overlaid two shapefiles in ArcGIS software intersecting the New Mexico Tribal lands shapefile with the zip code shapefile on to create a variable indicating whether the zip code includes any Tribal lands (see Figure 2). If the zip code contained any Tribal lands, it is coded as 1 (coded as 0 if the zip code did not contain any Tribal lands). If the zip code shared the boundary with a Tribal land, but did not include any territory, the Tribal land status of the zip code is coded as 0. Lastly, we reverse-geocoded the locations of abandoned uranium mines from the New Mexico uranium mines shapefile originating from the U.S Geological survey from the U.S. Department of the Interior (U.S. Department of the Interior, 2011), and aggregated the numbers of abandoned uranium mines to the zip code level. Presence of abandoned uranium mines is a dichotomous variable indicating whether or not the zip code includes any abandoned uranium mines.

### Data Analysis

There were no missing values. Out of 372 standard zip codes in New Mexico (i.e., zip codes that have physical locations; excluding P.O. Box zip codes that do not contain any physical areas)^2^, seven zip codes were excluded because there was no population. The final analytic sample included 366 zip codes. We ran three sets of ordinary least square (OLS) regressions: Model 1 includes all four dimensions of vulnerability by the SVI: socioeconomic status vulnerability, housing composition & disability vulnerability, minority status & language vulnerability, and housing and transportation vulnerability variables. Model 2 adds COVID-19 related vulnerability variables to the SVI, and the final model adds historically-embedded vulnerability variables (Model 3). Consistent with previous research (Karaye and Horney, 2020), we exponentiated the model coefficients for ease of interpretation, except for two dichotomous variables.

## Results

Table 1 shows the descriptive statistics of all variables, by total and Tribal land status. Our results demonstrate that there are significant and substantial differences in the confirmed COVID-19 cases by Tribal land status at the zip code level. On average, the rate of confirmed COVID-19 cases per 1,000 population for Tribal lands was 22.33 whereas it was 5.68 for non-Tribal lands. That is, the zip codes that contain Tribal lands experience nearly 4.5 times higher rates of COVID-19 cases (see Table 1). Characteristics of social vulnerability differed in all dimensions in which zip codes that include Tribal lands had significantly more socioeconomic status vulnerability (i.e., higher percentages of population living below poverty and unemployed) and more household composition & disability vulnerability (i.e., higher percentages of population under 18 and over 65, with a disability; and a higher percentage of single-parent household). Zip codes that include Tribal lands also had significantly higher percentages of race and ethnic minority population, living in crowded housing and without a vehicle. Similarly, zip codes that include Tribal lands had higher population density and percentage of population without health insurance. Lastly, the characteristics of historically-embedded vulnerability differed significantly in which zip codes that include Tribal lands had significantly higher percentages of households without telephone, Internet, and complete plumbing. For example, about 5.58% of households in zip codes including Tribal lands do not have complete plumbing compared to 1.25% of household in zip codes containing no Tribal lands.

**Table 1:** Descriptive statistics by Tribal land status.

	**Total** ** (*****n*** **=** **365)**		**Tribal land** ** (*****n*** **=** **125)**			**Non-Tribal land** ** (*****n*** **=** **240)**	
	**m/%**	**std**	**m/%**	**std**		**m/%**	**std**
**Confirmed COVID-19 cases per 1,000**	11.38	27.14	22.33	38.78	***	5.68	15.69
**Socioeconomic status vulnerability**							
Percent below poverty	18.66	17.48	21.92	16.03	*	16.96	18.00
Percent unemployment	7.18	11.28	9.52	9.48	**	5.96	11.95
Logged per capita income	9.98	0.47	9.90	0.48	*	10.02	0.46
Percent without a high school diploma	16.56	16.65	15.90	11.21		16.90	18.89
**Household composition and disability vulnerability**							
Percent children under 18	19.06	11.96	21.81	8.91	***	17.63	13.06
Percent elders 65 and older	23.16	18.17	19.72	11.06	**	24.95	20.73
Percent with a disability	19.65	14.41	17.80	9.98	*	20.61	16.18
Percent of single-parent household	31.20	27.19	40.66	22.26	***	26.28	28.24
**Minority status and language vulnerability**							
Percent racial and ethnic minority	27.33	31.70	40.75	37.14	***	20.34	25.91
Percent speak English less than “well”	3.89	8.41	2.60	3.56	*	4.56	9.99
**Housing and transportation vulnerability**							
Percent large apartment buildings	1.45	4.30	1.31	4.16		1.53	4.38
Percent mobile homes	27.77	20.41	26.26	16.41		28.55	22.20
Percent crowding	4.34	8.34	6.88	7.33	***	3.02	8.54
Percent without a vehicle	5.45	8.47	6.98	6.62	**	4.65	9.20
Percent living in group quarters	2.76	11.90	2.01	9.38		3.15	13.03
***COVID-19 related*** **vulnerability**							
Logged population density	2.31	2.59	3.01	1.98	***	1.95	2.79
Percent without insurance	12.04	13.73	15.74	14.62	***	10.12	12.86
***Historically-embedded*** **vulnerability**							
Percent without telephone	3.98	8.01	5.42	6.46	**	3.23	8.62
Percent without Internet	37.13	24.94	41.62	26.88	**	34.79	23.59
Percent without complete plumbing	2.73	6.69	5.57	8.73	***	1.25	4.71
Tribal land status (yes or no)	0.34	0.48	1.00	0.00		0.00	0.00
Presence of abandoned uranium mines	0.25	0.43	0.34	0.48	***	0.20	0.40

* *p < 0.05*,

** p < 0.01, and

*** *p < 0.001*.

Table 2 presents the results of OLS regression models to assess the relative impacts of various indicators of social vulnerability and structural inequalities from historical injustices on confirmed COVID-19 cases in New Mexico. Prior to building the final saturated model, we added each set of vulnerability characteristics one at a time to examine their contribution to the exploratory power and found that the historically-embedded vulnerability dimension had the greatest explanatory power of the variations in the rates of confirmed COVID-19 cases. Model 1 includes all 15 variables from the CDC's SVI. Variables from the SVI account for explaining about 25.8% of the variance in the rates of confirmed COVID-19 cases. Of the CDC's SVI characteristics, the percentages of population without a high school diploma and racial/ethnic minorities along with percentages of housing units with crowded housing (i.e., more than one person per room) and without a vehicle were associated with higher COVID-19 rates. For example, a one percent increase in percent racial and ethnic minority is associated with a 22% increase in the confirmed COVID-19 when all other variables are held constant.

**Table 2:** OLS exponentiated regression results predicting confirmed COVID-19 cases per 1,000.

	**Model 1**		**Model 2**		**Model 3**	
**Socioeconomic status vulnerability**						
Percent below poverty	1.06		1.07		0.88	
Percent unemployment	0.96		1.00		0.86	
Logged per capita income	0.01		0.05		0.32	
Percent without a high school diploma	1.34	*	1.32	*	1.19	
**Household composition and disability vulnerability**					
Percent children under 18	1.01		1.09		1.07	
Percent elders 65 and older	1.01		1.07		1.07	
Percent with a disability	0.94		0.95		0.90	
Percent of single-parent household	0.99		0.98		0.93	
**Minority status and language vulnerability**						
Percent racial and ethnic minority	1.22	***	1.21	***	1.09	*
Percent speak English less than “well”	0.76		0.71		0.82	
**Housing and transportation vulnerability**						
Percent large apartment buildings	1.05		1.10		1.56	
Percent mobile homes	1.01		1.01		1.11	
Percent crowding	2.03	***	1.88	***	1.53	**
Percent without a vehicle	1.67	*	1.69	*	1.03	
Percent living in group quarters	0.98		1.07		1.09	
***COVID-19 related*** **vulnerability**						
Logged population density			1.06		1.79	
Percent without insurance			1.38	**	1.27	*
***Historically-embedded*** **vulnerability**					1.00	
Percent without telephone					2.57	***
Percent without Internet					1.04	
Percent without complete plumbing					4.23	***
Tribal land status (yes or no)					5.92	*
Presence of abandoned uranium mines					2.17	
Constant	48.07		19.92		2.52	
*R*-squared	0.258		0.272		0.405	

* *p < 0.05*,

** p < 0.01, and

*** *p < 0.001*.

When the COVID-19 related vulnerability variables are added (Model 2), the significant associations of SVI indicators remain constant; and percentage of population without health insurance is positively associated with the rates of confirmed cases. In the final saturated model (Model 3), once historically-embedded vulnerability variables are added, the significant associations of percentages without a high school diploma (i.e., socioeconomic status vulnerability) and without vehicle disappeared. Percentages of house units without telephone and complete plumbing were significantly associated with rates of confirmed COVID-19 cases. The presence of abandoned uranium mines is associated with a substantially higher rate of confirmed COVID-19 cases, 2.17 times higher than zip codes without abandoned uranium mines, although the relationship was not statistically significant. Accounting for all other indicators of vulnerability, Tribal land status was associated with 5.92 times higher rates of confirmed COVID-19 cases compared to zip codes that do not contain any Tribal lands. More importantly, adding indicators of historically-embedded vulnerability increased the exploratory power to 40.5% from 25.8% in Model 1.

## Discussion and Conclusion

Since the early phase of the pandemic, a substantial amount of attention has been paid to the disproportionate impacts of COVID-19 on Indigenous Peoples, especially those on Tribal lands (Kakol et al., 2020). However, due to long-standing and systematic erasure of Indigenous representation in data and race misclassification (Kukutai and Taylor, 2016; Yellow Horse and Huyser, 2020) of Indigenous Peoples during COVID-19; assessing the reliable and accurate impacts of COVID-19 on Indigenous Peoples and communities has been limited. While a recent study documents that the cumulative incidence of COVID-19 among AIAN Peoples were nearly 3.5 times higher compared to non-Hispanic white individuals (Hatcher et al., 2020); this estimation is based on only laboratory-confirmed COVID cases from 23 states. While it is an important study to help quantify the impacts of COVID-19 for AIAN Peoples, it is likely a gross under-estimation. Considering the serious data challenges, public health researchers and policy makers utilized the CDC's SVI to implement place-based COVID-19 mitigation efforts (Amram et al., 2020; Gaynor and Wilson, 2020; Kim and Bostwick, 2020); yet we identified that whether and how SVI can help mitigation efforts for Indigenous Peoples and communities is unclear.

There were several key findings of the study. First, we found that only selected indicators of the SVI were significantly associated with COVID-19 cases in New Mexico: percent of population who are racial/ethnic minority and percent of housing crowded housing units. Socioeconomic status vulnerability and household composition & disability vulnerability factors were largely not significant. This finding suggests that inability to practice quarantine in one's home due to crowding may contribute to spread of COVID-19 if one family member becomes infected. Thus, providing out-of-home safe quarantine space for those with COVID-19 can potentially prevent the spread of COVID-19. We interpret the significance of percent of population who are racial/ethnic minority as a proxy measure of systematic racism, and not as any inherent biological differences (Roberts and Rollins, 2020). Second, somewhat unsurprisingly, percent of population without health insurance was a significant factor associated with COVID-19 cases. This suggests that lack of access to health insurance should be explicitly acknowledged as a critical barrier for testing; and over-restriction should be adjusted for “critical” populations (e.g., front-line health care workers, essential workers, symptomatic patients, co-morbid populations) to get tested for both viral load (virologic testing) and antibodies (serologic assessment) (Pettit et al., 2020). Third, we found that higher percentages of house units without telephone and complete plumbing were associated with higher COVID-19 rates, similar to previous findings on the importance of households with lack of plumbing (Rodriguez-Lonebear et al., 2020) as well as challenges of telemedicine implementation in Tribal communities during COVID-19 (Graves et al., 2020).

The primary implication of our findings is the critical importance of historically-embedded vulnerability variables. Guided by Hózhó wisdom of Navajo people that emphasize the importance of interconnectedness and whole-system (Powell and Curley, 2008; Kahn-John and Koithan, 2015), we moved beyond simply considering social vulnerabilities defined by the CDC's SVI to look at factors that are important for Indigenous Peoples and communities. We found that historically-embedded vulnerability variables had the *largest* explanatory power compared to other social vulnerability factors from the SVI and COVID-19, especially Tribal land status. There are significant and substantial differences in the confirmed COVID-19 cases by Tribal lands status at the zip code level, and nearly all vulnerability characteristics varied significantly by Tribal land status. This finding illustrates that Tribal land status is a critical proxy measure of systematic racism against Indigenous Peoples that continue to have lasting implications on health of Indigenous Peoples and communities both directly and indirectly. Tribal lands signify physical manifestations of the lasting legacy of treaty violations impacting Indigenous Peoples and communities through lack of systematic funding investment in Tribal public health and household infrastructures (Rodriguez-Lonebear et al., 2020), and impact of displacement through settler colonialism. At the same time, Tribal lands are often sacred places where Indigenous resilience and healing take place. Emerging evidence suggests that non-Tribal governments' infringement on Tribal sovereign rights during the pandemic hurt Indigenous Peoples and communities (Hoss and Tanana, 2020). It highlights the urgent need to respect Tribal sovereign legal authority to respond to the needs of their communities. For example, the funding from the Coronavirus Aid, Relief, and Economic Security (CARES) Act must lift the restrictions on how and when the funds can be used; and respect the Tribal sovereign legal authority to use the funding to address the specific needs of Indigenous communities (Hoss and Tanana, 2020).

Although presence of abandoned uranium mines was not statistically significant, it had substantial impact on COVID-19 rates in our results. We urge future studies to investigate the role of abandoned uranium mines on health of Indigenous Peoples and communities during COVID-19 more systematically. Specifically, uncovering the mechanisms of indirect effects from abandoned uranium mines (e.g., access to safe water, levels of toxin from the abandoned uranium mines, etc.) can contribute to the literature documenting the lasting deleterious effects of environmental racism against Indigenous Peoples. This is extremely timely as President Trump's nuclear energy plan suggested the possibility of uranium mining near the Grand Canyon (Krol, 2020), lands that are sacred to many Indigenous Peoples including the Havasupai Tribe, Hopi Tribe, Hualapai Tribe, Kaibab Band of Paiute Indians, Las Vegas band of Paiute Indians, Moapa Band of Paiute Indians, Navajo Nation, Paiute Indian Tribe of Utah, San Juan Southern Paiute Tribe, The Pueblo of Zuni, and Yavapai-Apache Nation (National Park Service, 2019).

There are several limitations of the study. First, the study is an ecological assessment at the zip code level, and the findings cannot be inferenced to AIAN persons (e.g., ecological fallacy). If and when data is available at the individual level, future research must investigate the potential contextual influences of social and historically-embedded vulnerabilities on individuals. However, in New Mexico, about 73.8% of individuals residing on Tribal lands were AIAN persons (U.S. Census, 2019). Second, zip code as a unit of analysis may not reflect meaningful boundaries of Indigenous communities as zip code is designed for efficient delivery of U.S. postal mail. Future studies should assess whether and how the use of different administrative units may yield comparable results. Third, many Indigenous Peoples reside in households without a physical home address and use a P.O. Box, and omitting those who reside in households without a zip code may contribute to *underestimation* of impacts of COVID-19 as they are systemically erased from federal data through inaccurate and unreliable estimates of Indigenous Peoples (Kukutai and Taylor, 2016). Lastly, we do not capture potential inter-Tribal differences in New Mexico; and our findings may not be generalizable to other Tribal nations in different states.

Despite these limitations, our results from innovative data provide important insights for the place-based mitigating efforts and suggestions for the potential change for praxis. Specifically, our results suggest the needs for increasing out-of-home safe quarantine spaces, eliminating lack of health insurance as a barrier for testing, and explicit consideration of Tribal land status and infrastructure conditions, such as plumbing and information communication technology, in understanding the impacts of COVID-19. Furthermore, our results demonstrate the need for greater efforts to understand the experiences of Indigenous Peoples during COVID-19 from the social determinants of health framework (Kakol, Upson and Sood 2020) that explicitly incorporates historical racisms, but also from the Indigenous research framework that centers Indigenous knowledge and methodologies (Smith, 2013).

Given the disproportionately high COVID-19 confirmed cases and deaths among AIAN individuals (New Mexico Department of Health, 2020) and relatively small population size of AIAN persons at the aggregate level on non-Tribal lands; we urge public health researchers and policy makers interested in designing any place-based mitigating strategies to explicitly include percentage of AIAN Peoples as one of the main factors of identifying vulnerable places rather than the percentage of racial and ethnic minority. This is critical, without careful attention on whom we exclude through categorization and aggregation, any mitigating efforts could further harm Indigenous Peoples and communities who are often already marginalized through inadequate and unjust representation in federal data (Taylor, 2009; Kukutai and Taylor, 2016). Any research and public health policies with AIAN Peoples that do not explicitly consider the Indigenous research framework will likely produce erroneous conclusions and/or strategies for Indigenous Peoples; and that will contribute to further harming AIAN Peoples and communities.

## Data Availability Statement

The original contributions presented in the study are included in the article/Supplementary Material, further inquiries can be directed to the corresponding author.

## Author Contributions

All authors participated in the formulation of the research questions and writing. AYH is responsible for the data analysis. All authors contributed to the article and approved the submitted version.

## Conflict of Interest

The authors declare that the research was conducted in the absence of any commercial or financial relationships that could be construed as a potential conflict of interest.

## Footnotes

^1^See the National Congress of American Indians (NCAI) Response to Usage of the Term, “Indian Country”: https://www.ncai.org/news/articles/2019/12/27/ncai-response-to-usage-of-the-term-indian-country.

^2^The contextual-level characteristics of zip code *areas* derived from information from households that reside within the zip code areas, and P.O. Box zip codes do not contain any residents as they are often the addresses of physical locations where the mail boxes are located.
